# Community members and healthcare workers’ priorities for the control and prevention of snakebite envenoming in Ghana

**DOI:** 10.1371/journal.pntd.0011504

**Published:** 2023-07-21

**Authors:** Leslie Mawuli Aglanu, John Humphrey Amuasi, Evie Prokesh, Alexis Beyuo, Chrisantus Danaah Dari, Sofanne J. Ravensbergen, Melvin Katey Agbogbatey, Austin Gideon Adobasom-Anane, Kabiru Mohammed Abass, David G. Lalloo, Jörg Blessmann, Benno Kreuels, Ymkje Stienstra

**Affiliations:** 1 University Medical Centre Groningen, Department of Internal Medicine/Infectious Diseases, University of Groningen, Groningen, The Netherlands; 2 Global Health and Infectious Diseases Research Group, Kumasi Centre for Collaborative Research in Tropical Medicine, Kumasi, Ghana; 3 Department of Global Health, School of Public Health, Kwame Nkrumah University of Science and Technology, Kumasi, Ghana; 4 Department of Development Studies, Simon Diedong Dombo University of Business and Integrated Development Studies, Upper West Region, Wa, Ghana; 5 Regional Health Directorate, Ghana Health Service, Upper West Region, Wa, Ghana; 6 Research Group Snakebite Envenoming, Department of Implementation Research, Bernhard Nocht Institute for Tropical Medicine, Hamburg, Germany; 7 Presbyterian Hospital, Agogo, Ashanti Region, Ghana; 8 Centre for Snakebite Research and Interventions, Liverpool School of Tropical Medicine, Liverpool, United Kingdom; 9 Division for Tropical Medicine, Department of Medicine, University Medical Centre Hamburg-Eppendorf, Hamburg, Germany; The University of Sydney School of Veterinary Science, AUSTRALIA

## Abstract

**Introduction:**

Snakebite is one of the most neglected tropical diseases. In Ghana, there has been a limited interest in snakebite envenoming research despite evidence of high human-snake conflicts. In an effort to meet the World Health Organisation’s (WHO) 2030 snakebite targets, the need for research evidence to guide policy interventions is evident. However, in setting the research agenda, community and healthcare workers’ priorities are rarely considered.

**Methods:**

Three categories of focus groups were formed in the Ashanti and Upper West regions of Ghana, comprising of community members with and without a history of snakebite and healthcare workers who manage snakebite patients. Two separate focus group discussions were conducted with each group in each region. Using the thematic content analysis approach, the framework method was adopted for the data analysis. A predefined 15-item list of potential snakebite-associated difficulties and the WHO’s 2030 snakebite strategic key activities were ranked in order of priority based on the participants’ individual assessment.

**Results:**

Both acute and chronic effects of snakebite such as bite site management, rehabilitation and mental health were prioritised by the community members. Health system challenges including training, local standard treatment protocols and clinical investigations on the efficacy of available antivenoms were identified as priorities by the healthcare workers. Notably, all the participant groups highlighted the need for research into the efficacy of traditional medicines and how to promote collaborative strategies between traditional and allopathic treatment practices.

**Conclusion:**

The prioritisation of chronic snakebite envenoming challenges by community members and how to live and cope with such conditions accentuate the lack of post-hospital treatment follow-ups for both mental and physical rehabilitation. To improve the quality of life of patients, it is essential to involve grassroots stakeholders in the process of developing and prioritising future research agenda.

## Author summary

The WHO strategy for the prevention and control of snakebite envenoming identified community engagement and empowerment as a key pillar in meeting the target of halving death and disability by 2030. However, community members’ priorities are rarely considered in the design of health research and treatment approaches. This study focussed on describing the needs, experiences and priorities of community members and healthcare workers in two high snakebite prevalence regions in Ghana. Our findings reflect similar priorities to those identified by the WHO but also highlight other community needs that are not emphasised by the research community. The community members’ priorities include management of the bite site, pain, rehabilitation and mental health support whereas the healthcare workers prioritised training of health professionals, development of local standard treatment protocols and clinical investigations on the efficacy of antivenoms. Exploration of the efficacy of traditional medicines and the identification of traditional and allopathic synergistic treatment approaches were prioritised by both community members and the healthcare workers. Engaging and empowering communities in the research agenda setting process will help guide the conduct of priority research needed to improve the outcome and quality of life of snakebite victims.

## Introduction

Snakebite is a constant public health threat that affects low and middle-income countries (LMICs). The incidence of snakebite envenoming (SBE) is about 10 fold higher in rural areas than in urban areas [1]. In Ghana, the hospital visit rate of snakebite victims is estimated at 35/100,000 persons per year with up to 11% case fatalities reported in a pre-intervention study at a rural health facility [2,3]. A relative risk of disability has been estimated at 1.54 (95% CI, 1.17–2.03) in snakebite victims compared to the control group in the country [2]. Similar to other diseases of poverty, investment and attention to SBE have been minimal until recently. Over the years, SBE had been omitted from the list of research priorities by funding agencies, thereby attracting limited attention and interest among global health researchers and policymakers [4]. This has been largely attributed to the demographic category of the most affected populations and their lack of political voice [5,6].

Through the four strategic objectives outlined in the Strategy for Prevention and Control of Snakebite Envenoming, the World Health Organisation (WHO) targets a 50% decline in global mortality and disabilities due to snakebite by 2030 [7]. Since the launch of the strategy, there have been a growing interest and demand by decision-makers for quality research evidence to inform appropriate policies and interventions. However, among the major barriers to meeting the WHO targets in Sub-Saharan Africa are limited research evidence, misalignment of local priority needs and the lack of investment towards effective therapies targeting local snake species [1,5,6]. As attention to SBE increases, it is imperative to identify community-level priorities to guide the development of a research agenda that meets local needs.

Although patient and community involvement in health research is increasingly being advocated for, their priorities are often unexplored or left unaddressed [8,9]. Compared to professionals and researchers, community and patients’ priorities are sometimes different and often dismissed in the process of health research agenda setting [10–12]. Departing from specific research questions about the effects of treatment, ulcerative colitis patients in the UK, for instance, placed a high priority on research that focuses on identifying how to live and cope with their condition [13]. In an effort to improve the integrated management of skin neglected tropical diseases (NTDs) in Nigeria, community members expressed interest in how to undertake self-care practices [14]. In rural South Africa, community members raised concerns about treatment fatigue, the size of tablets and the side effects of tuberculosis medication [15]. Similar sentiments were shared about efforts towards reducing the side effects of lymphatic filariasis medication in communities with persistent transmission of lymphatic filariasis in Ghana [16]. These findings provide evidence that patients and community members do sometimes prioritize different aspects of treatment in comparison to the priorities of the international research communities. The bedside experiences and needs of healthcare workers who contribute to the treatment and management of snakebite patients are equally essential to the health research agenda setting. In Ghana, healthcare workers in rural and peri-urban settings were found to have below average knowledge of snakebite management [17]. Similar knowledge and need gaps in diagnostics, prescription and administration of antivenom, and the identification of venomous snake species have been reported among various healthcare professionals in other LMICs [18–21].

The available research evidence postulates possible health outcomes and quality of life improvement opportunities by synergising the priorities of patients, community members and clinicians with that of researchers and policymakers. Focusing on their lived experiences, this study engaged community members with and without a history of snakebite and healthcare professionals who take care of snakebite patients in Ghana to identify their snakebite research priority needs. The emerging priorities will be compared to the WHO SBE strategic objectives.

## Methods

### Ethics statement

Ethical approval was granted by the Ghana Health Service Ethics Review Committee (GHS-ERC010/03/20) and the Medical Ethical Committee of the University Medical Centre Groningen, The Netherlands (NL-LTc201900047). All participants voluntarily provided a signed or thumb printed informed consent after the content of the information sheet and consent forms were read and understood. If the participant could not read and understand English, a translation to a local language such as Twi or Waale was provided based on their preference. The community health volunteers acted as witnesses in the consenting process of the community members.

### Study participants

Three categories of focus groups comprising community members with and without a history of snakebite and healthcare workers who contribute to the treatment of snakebite patients were engaged through the consultation, prioritisation and integration phases of the research agenda setting process [22,23]. The focus groups were consulted separately through group discussions at the hospital and community levels between September and October 2019, and February 2021 in the Ashanti and Upper West regions of Ghana respectively. The timing of data collection was influenced by the global COVID-19 pandemic and its associated travel and community engagement restrictions. The Ashanti and Upper West regions were selected on account of the high number of health facility-reported snakebite cases discovered in our preliminary analysis of data obtained from the Ghana District Health Information Management System (DHIMS).

In consultation with local collaborators and the hospitals’ administrative staff, participants of the healthcare workers’ focus group discussions were identified through purposive sampling in the Wa Municipal Hospital which until 2019 served as the Upper West regional referral hospital. Although it is now the Wa municipal hospital, it still receives high numbers of snakebite cases including referrals from other health facilities in the region. In the Ashanti region, participants were sampled from the Agogo Presbyterian Hospital which serves as the referral hospital in the Asante-Akim North municipality of the Ashanti region. The sampling of healthcare workers to participate in the discussion was based on their active engagement in the treatment and management of snakebite victims for at least one year within the health facility. The invited professionals consisted of clinical pharmacists, physicians, physician assistants, nurses, environmental health officers, physical therapist assistants and anaesthesiologists working in the various wards including the medical ward, emergency unit, children/paediatric ward, physiotherapy department and public health departments.

In the other two categories of focus groups, the research team with support from local collaborators and the community health volunteers invited community members with a history of snakebite into one group and community members without a history of snakebite into another group through purposive sampling. In the Upper West region, we sampled two communities based on the number of snakebite cases reported at the Wa municipal hospital within the previous two years. In the Ashanti region, participants were invited to the hospital from the catchment communities served by the Agogo Presbyterian Hospital due to the lack of access to detailed data on communities where snakebite victims reside. Patients reporting to the hospital for various conditions apart from snakebite were also invited to participate in the focus group discussions as community members after they were treated and discharged. Community members below the age of 18 were excluded. We aimed to recruit between six and ten participants for each of the twelve focus group discussions.

### Data collection

Two focus group discussions were held with each of the three stratified groups in each region. The group discussions with healthcare workers were conducted at the conference halls of both hospitals. The discussions with community members were held in the halls of the community health centres in the Upper West region and the conference hall of the Agogo Presbyterian Hospital in the Ashanti region. The discussions were conducted in English with the healthcare workers but translated from English to the local languages (Twi or Waale) and vice versa between the interviewer, the interpreter and the community participants. A semi-structured interview guide covering the local context of the burden of snakebite, presentation at health facilities, diagnosis, treatment and rehabilitation, healthcare workers’ competence and community engagement was used to direct the discussions. The semi-structured interview guides were tailored to each of the three stratified groups. All the focus group discussions were audio-recorded and lasted about 90 minutes each.

At the end of the focus group discussion with community members with a history of snakebite and healthcare workers, the groups deliberated on a 15-item list of potential challenges that could be associated with snakebite at the community level. The list was predefined based on the authors’ previous field experiences with community members and healthcare workers, and evidence from a study on former Buruli ulcer patients where victims displayed chronic physical and psychosocial effects akin to SBE [24]. Each participant was given the 15-item list and tasked to individually rank each item based on how challenging or difficult it is using a 5-point Likert scale i.e., “I don’t know”, “extremely difficult”, “very difficult”, “a little bit difficult”, and “not difficult at all”.

A list of the WHO strategic objectives on halving SBE deaths and disability by 2030 and their associated key activities were provided to the healthcare workers. Considering each key activity as a globally coordinated response to SBE, the healthcare workers were asked to rank these activities from “first” being the most urgent activity to “sixth” being the least urgent activity to be implemented. The percentage score for each rank of each activity was weighted; the “first” was weighted by 6 and the “sixth” was weighted by 1. The average weighted scores were used to rank the key activities under each of the WHO strategic objectives in order of priority.

### Data analysis

Using the framework method [25,26], a thematic content analysis approach was employed for the qualitative data analysis [27]. This method is a systematic and verifiable approach to identifying emerging similarities and differences and allows researchers to draw clustered descriptive findings based on the developed themes [28,29]. Analysis of the transcripts was carried out by two researchers who read all the transcripts and developed a framework of codes. The codes were organised based on the key activities identified by the WHO under the snakebite strategic objectives after discussing and merging the independently generated codes to develop the framework. ATLAS.ti version 22 software was used for the data analysis. The resulting codes and themes were compared for inter-coder reliability. The narratives of each group discussion were indexed, charted and triangulated with each other to identify both common and divergent themes and the significance and priority of the emerging themes.

The demographic data (region, focus group category, gender, age), the 15-item list of potential challenges mostly associated with snakebite, and the ranked activities of the WHO SBE strategic objectives were analysed using descriptive statistics. All the descriptive statistical analyses were performed with IBM SPSS Statistics version 26.

## Results

### Participant characteristics and priority rankings

A total of 12 focus group discussions comprising of 85 participants were conducted for this study. Two separate focus group discussions were held with each of the three focus group categories in each study region. Males made up 65.9% of the participants. The median (IQR) age of the participants is 36 (32–46) (Table 1).

**Table 1:** Demographic characteristics of the study participants.

	Snakebite Victims n (%)	Community members n (%)	Healthcare Professionals n (%)
**Region**			
Ashanti	11 (35.5)	15 (50.0)	13 (54.2)
Upper West	20 (64.5)	15 (50.0)	11 (45.8)
**Gender**			
Male	23 (74.2)	19 (63.3)	14 (58.3)
Female	8 (25.8)	11 (36.7)	10 (41.7)
**Age** (years)			
Median (IQR)	37.0 (30.5–48.5)	35.0 (32.0–48.0)	35.0 (31.0–39.0)

**Fig 1:**
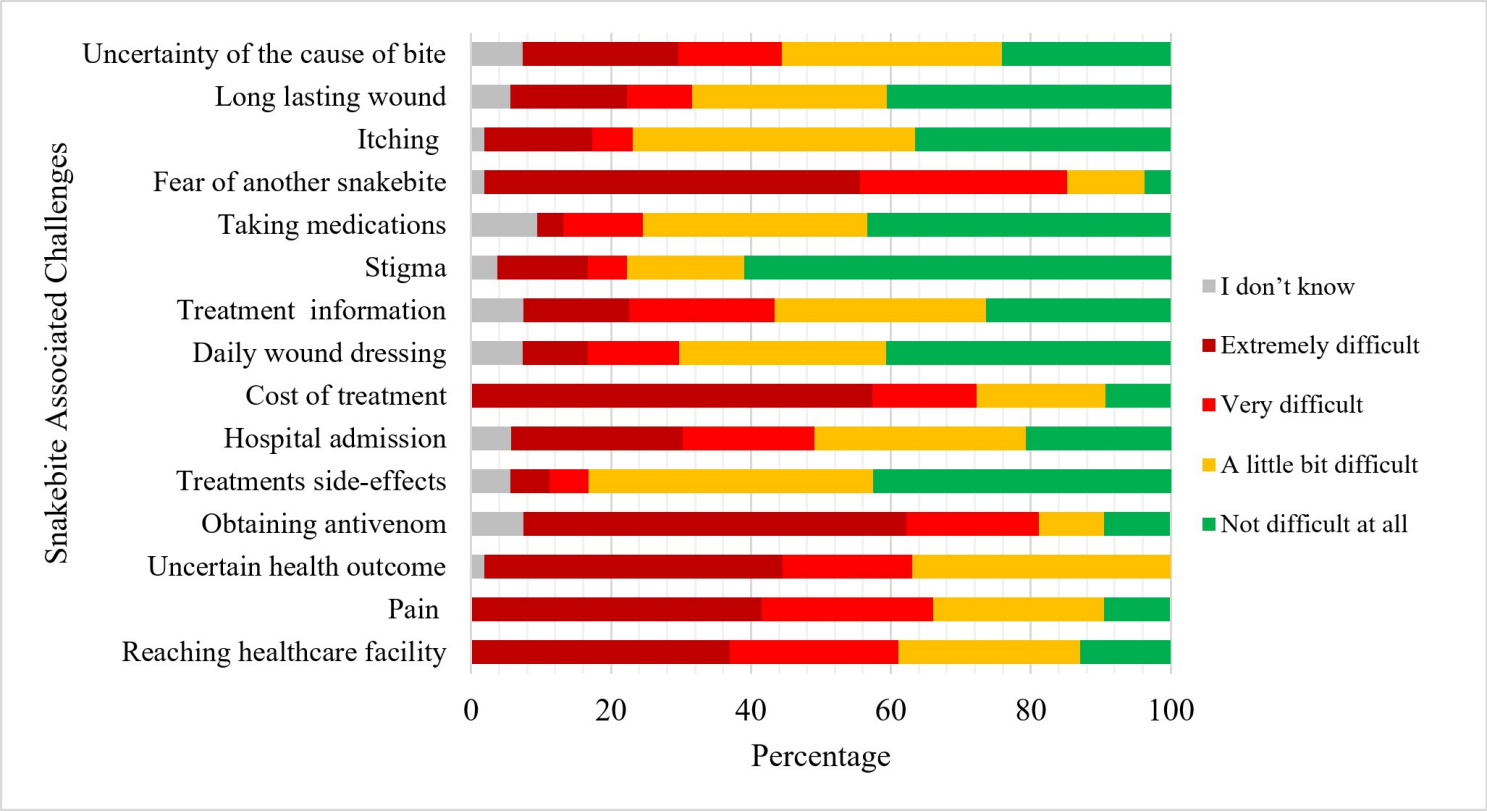
Community members with a history of snakebite and healthcare workers’ ranking of challenges associated with snakebite.

The healthcare workers who contribute to the treatment of snakebite patients and community members with a history of snakebite reflected on the degree of difficulty in handling each of the predefined challenges that could be associated with a snakebite. As presented in Fig 1, the most difficult-to-manage challenges identified are uncertainty about health outcomes, fear of another snakebite, pain associated with the bite and access to treatment related challenges (reaching healthcare facility, obtaining antivenom and cost of treatment). There were no significant differences between how the community members and the healthcare workers ranked the challenges associated with snakebite management.

**Fig 2:**
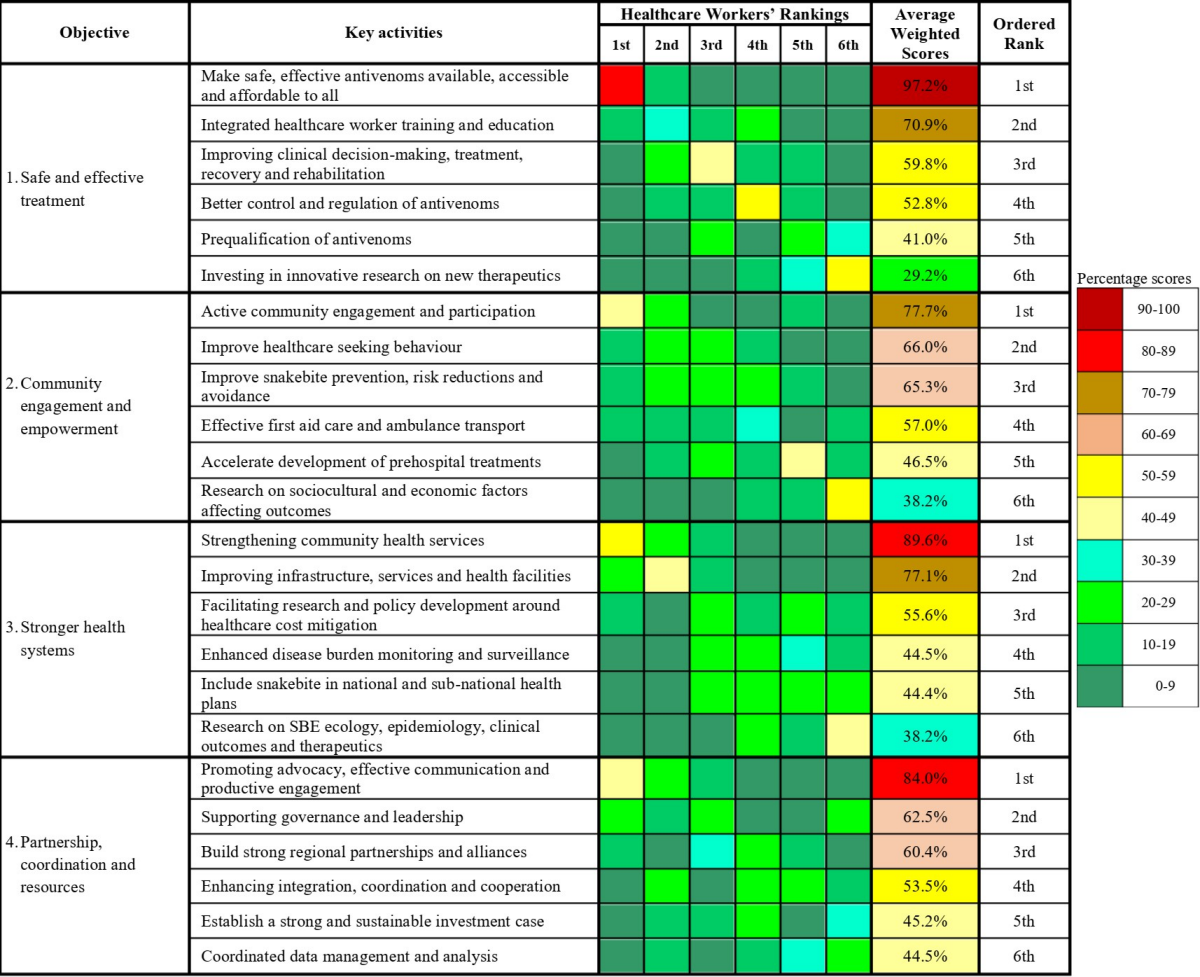
Healthcare workers’ ranking of the WHO SBE strategic objectives’ key activities.

The healthcare workers ranked the key activities under each of the WHO snakebite strategic objectives in order of priority. The average weighted score for each key activity shows the resulting priority implementation order (Fig 2). Under objective one, ‘making safe and effective antivenoms available, accessible and affordable to all’, and ‘integrating snakebite management into healthcare worker training and education’ were ranked as the top two priority activities. Under the second objective, three out of the six activities were prioritised. These are ‘actively engaging communities and improving participation in tackling snakebite’, ‘improving healthcare seeking behaviour’, and ‘improving SBE prevention, risk reductions and avoidance’ at the community level. Under the stronger health systems objective, the healthcare workers prioritised ‘strengthening community health services’, and ‘improving infrastructure, services and health facilities’. Under the fourth strategic objective, three key activities were prioritised. These are ‘promoting advocacy, effective communication and productive engagement’, ‘supporting governance and leadership’, and ‘building strong regional partnerships and alliances’.

### Emerging priorities from the focus group discussions

We engaged community members with and without a history of snakebite, and healthcare workers who contribute to the treatment of snakebite patients in two distinct geographic locations with rural and peri-urban characteristics in discussing the challenges associated with snakebite in their daily lives. Four major themes were developed based on the WHO strategic objectives and applied to the focus group discussions.

### Safe and effective treatment

Access to effective and affordable treatment was identified as the first research priority need among all the focus groups. Participants of the focus group discussions highlighted that although free antivenom is supplied monthly by the government through the regional medical stores to designated health facilities, the quantity is insufficient to meet the demand. The healthcare workers indicated that some health facilities procure more vials from their internally generated funds and offer them to patients at a fee, refer patients to other facilities or write a prescription for patients to procure antivenoms at private pharmacies when the stock from the government runs out. At the time of conducting this study, the average cost per vial of antivenom was estimated at 87 USD in private pharmacies. Unfortunately, the hospital staff and family efforts are not always successful in securing antivenom but data on mortality rates resulting from the lack of antivenoms in health facilities are not available.

*“The most disturbing part is for you to be bitten by a snake*, *only to be rushed to the hospital and there is no antivenom available*.*”* Male, community members without snakebite history FGD 1

*“Another thing we can also do to help the victims is to totally prioritise and make the management of snakebite totally free*.*”* Male nurse, healthcare workers FGD 3

The quality of antivenom in the market was also questioned by all three focus group categories. Some of the healthcare workers indicated that the scarcity of quality antivenoms forces them to use available but unapproved/ineffective antivenoms, some of which do not target local snake species. Calls were made for the Food and Drugs Authority (FDA) to stringently enforce regulation on the quality of antivenoms in the market, including appropriate storage to maintain their efficacy.

*“There are some types when you use*, *it works fast but there are others you will use more than 10 [vials]*. *So we can’t tell actually what is causing that”* Male nurse, healthcare workers FGD 4

*“Some people also go to buy some ASVs from other areas to come for administration but some are usually identified as not FDA approved*.*”* Male physician, healthcare workers FGD 3

New and reliable diagnostic procedures were highlighted as urgently needed for appropriate diagnosis of snakebite, especially in cases where patients come in with an unknown bite. Calls were also made by the healthcare workers for more research into reducing anaphylaxis and other side-effects such as nausea, vomiting, dry cough, itchiness, chills and drowsiness, and for exploring the potential of using herbal medicine for snakebite management.

*“With the current one [antivenom] the hospital is providing*, *usually what we have observed is that between 80–90% react to the brand we are using*. *Usually the sign we observe is extreme chills*.*”* Male nurse, healthcare workers FGD 3

All the healthcare workers highlighted the importance of post-qualification training including how to conduct and record bedside clotting test and the general clinical management of SBE including indications for antivenom and its safe administration. None of the participants in the discussion had received any post-qualification training on snakebite management. One of the participants suggested target specific training and workshops for all healthcare workers, especially those in rural areas. The training was further recommended to be integrated into available continuous professional development courses to encourage participation.

*“At times too we are too much in a hurry to give the anti-snake venom*. *Maybe some snakebites may not come with envenomation but we are too much in a hurry*. *So*, *I think we need to be cautious of that*.*”* Male physician, healthcare workers FGD 1

*“Snakebite is an emergency so you can’t say you have enough knowledge… we always have new professionals joining so training is key*.*”* Female physician, healthcare workers FGD 3

As part of efforts to improve recovery and rehabilitation, patients are often asked to report back to the health facility if they develop any further symptoms or complications after discharge. However, the majority of the participants with a history of snakebite did not return. Those who developed further symptoms or complications often preferred to use traditional medicine until it proves ineffective. The healthcare workers confirmed that less than 5% of victims who are asked to return for review after discharge do make it for the appointment. The common post-discharge symptoms and complications mentioned include post-traumatic stress disorder, swelling of the affected limb, weak limb, abnormal chills, contractures and psychosocial impact. Calls were made by the participants with a history of snakebite to improve the treatment of both acute and chronic effects of snakebite.

*“I don’t know much but the doctors should find a way that can improve the healing [participant with contracture on the fingers]*.*”* Male, community members with snakebite history FGD 3

*“We feel that if the hospital could give us some medicine for further treatment so that it can solve that problem of getting constant chills during raining season*.*”* Male, community members with snakebite history FGD 4

*“Probably the psychological aspects*. *They will be living with a lot of fear and anxiety*, *post-traumatic stress disorder*.*”* Male physician, healthcare workers FGD 1

### Community engagement and empowerment

All the participants acknowledged the potential impact of community engagement and sensitisation but highlighted the need for more targeted and community-oriented strategies. Some of the community members asserted that they have never received or participated in any form of snakebite community engagement activity but are willing to participate if such activities are appropriately designed. A few were pessimistic about the effects of preventive strategies, referring to the chances of being bitten as destined.

*“For me*, *had it not been for today’s discussion where you are talking about how to protect ourselves from snakebites*, *I have never heard anyone talk about this issue*.*”* Male, community members without snakebite history FGD 3

*“We need education on prevention as well as after treatment what we are supposed to do so that we will fully recover*.*”* Male, community members with snakebite history FGD 3

*“I don’t think sensitization can help in any way because snakes live in the bush and you cannot tell when you will encounter one*. *So I think it is only God that can protect us*.*”* Female, community members with snakebite history FGD 1

The community members largely agreed that all snake species possess some form of venom but did acknowledge the difficulty in identifying the “more venomous” snake species prevalent in the region. Identification of venomous snake species was mostly based on personal and other people’s experiences. Uni-coloured and smooth skin looking snakes were deemed as less or non-venomous.

“*The venomous ones are most likely to be multi-coloured and rough and the non-venomous ones are those that are very smooth … when the venomous one bites it leaves the fangs in the person so we have to remove the fangs*.*”* Male, community members without snakebite history FGD 3

The healthcare workers highlighted that community members often practised adverse first aid measures including ingesting various substances, sucking out the venom, application of tourniquets, incisions to remove fangs and the application of herbal medicines on the bite site. Other community perceptions and traditional treatment practices mentioned include victims not sleeping for fear of death, ensuring the bite site does not come into contact with water and making incisions at other parts of the body, distant from the bite site, to apply medicine. The community members however did not question the safety and efficacy of such practices. The high patronage of the services of traditional healers in these regions suggests a need to explore collaborative treatment approaches between traditional and allopathic medicine. Some of the participants advocated for the need to scientifically evaluate traditional medicines for the presence of potential venom inhibitors that could be used in the development of new therapies.

*“We know the fangs are there through the traditional treatment that we use to do*.*”* Male, community members without snakebite history FGD 3

*“I went to the traditional healer to remove the fangs and he told me not to allow water touch the bite site*. *So when I was even showering I had to raise my leg to avoid contact with water*.*”* Female, community member with snakebite history FGD 2

*“There have been proven effective non-orthodox medications … better tolerated than the conventional medicines that we have in our system*. *But the point is*, *because of the tradition of handing over … there is no proper documentation and further research*.*”* Male pharmacist, healthcare workers FGD 3

Uncertainty of victims’ health outcome and fear of another bite were regarded as major concerns for victims by all the focus groups. Spiritual and superstitious connotations of snakebite were believed to lead to death if the victim is suspected to have committed a crime, offended the gods or been bewitched. The community members indicated a need to explore ways of overcoming this fear as it affects their daily livelihood activities.

*“It’s about the fear of death and fear of another bite because the person keeps thinking that if I engage in this I will get another bite*.*”* Male, community members without snakebite history FGD 3

*“Anxiety*, *fear of the unknown*, *whether they are going to die or not die*.*”* Male anaesthesiologist, healthcare workers FGD 1

*“We know some of the bites are as a result of spiritual issues like witchcraft … those that resulted from witchcraft or spiritual bites they don’t even survive*.*”* Male, community member with snakebite history FGD 3

### Stronger health systems

As a medical emergency, community members lamented the weak healthcare system, citing poor access, inadequate staffing and language barrier as some of the major challenges. All three focus group categories highlighted the need to explore ways of improving the administrative procedures at the hospitals in efforts to improve treatment efficiency.

*“When we reach the hospital they will ask us to go and take folder*, *you have to go and queue and collect the folder meanwhile they don’t have an idea of how long you have taken from your house or the place of the bite up to the hospital*.*”* Male, community members without snakebite history FGD 3

All the focus groups advocated for the strengthening and capacity building of community health centres to enable them to manage snakebite cases. Participants from all the focus groups argued that a high proportion of snakebite cases occur in rural communities but antivenoms are stocked in the cities and often out of reach of those who need them the most. The development of standardised treatment protocols including pre-hospital care and their deployment in rural health facilities was suggested by the healthcare workers with the hope that it could promote effective and appropriate first aid practices at the community level before patients are referred to higher level health facilities for advanced treatment.

*“We would like the antivenom to be provided here at the community health centre*. *If they make the antivenom available at the community health centres*, *when we get cases we will not waste time to travel …”* Male, community members without snakebite history FGD 3

*“Sometimes they [victims from rural communities] have to travel 3–4 hours… So if these drugs are available for them on their doorsteps … the health post there can manage them before they are even referred*.*”* Female physician, healthcare workers FGD 2

The healthcare workers noted that most of the health facilities lack appropriate health equipment including diagnostics and cold chains, and the required human resources and capacity. This results in the family of patients often being requested to donate fresh blood in cases when needed to augment treatment.

*“The handling of the bedside clotting is a challenge*, *with the inappropriate tools that we are to use*, *it’s a challenge*.*”* Male nurse, health workers FGD 4

*“Sometimes they come in a complicated state and they may need fresh frozen plasma as well*. *And the hospital facility*, *housing a lot of fresh frozen plasma to support such cases is also another challenge*. *Most of the time we don’t get enough that would help supplement the platelet loss*.*”* Male nurse, healthcare workers FGD 2

Generating data on the disease burden through monitoring and surveillance was suggested by the healthcare workers as an approach that could increase government commitment to the implementation of control and preventive interventions. Emphasis was placed on generating research evidence on mortality, post-treatment symptoms including the frequently mentioned abnormal chills, functional limitations, mental health as well as healthcare workers’ training needs.

*“Even when we use ASV we assume that we are neutralizing the snake venom but there have been instances that people are discharged and they … come back with some side complications … literature is very limited in some of these areas*.*”* Male pharmacist, healthcare workers FGD 3

*“When you are a victim*, *during any rainy season you will have a problem of cold*. *And the site sometimes*, *most of them even get swollen again so they are always traumatised during cold weather*.*”* Female, community members without snakebite history FGD 4

### Partnership, coordination and resources

Discussion on partnerships and coordination activities was limited to the national and local contexts. The establishment of poverty alleviation programmes for victims who become incapacitated was suggested to improve their livelihood. For a more coordinated response, the integration of snakebite interventions into other health programmes and the basic education curricula was seen as a cost-effective approach to implementing snakebite programmes. However, poor collaborative partnership between the health and media sectors was identified to be a major drawback to the appropriate and timely dissemination of life-saving information.

*“If we also get some sort of support from government so that those that are victims and cannot probably go back to their work they get some support from government*.*”* Male, community member without snakebite history FGD 4

*“These days every programme tries to integrate another programme*. *So let’s say once the community health nurses are going there [for community outreach activities] … then they will also be sensitising them on snakebites as well*.*”* Male anaesthesiologists, healthcare workers FGD 1

*“If we can also include it in our basic curriculum in the schools … because when children get information it is easier for it to be transmitted to the general public*.*”* Female nurse, healthcare workers FGD 2

Healthcare workers including community and public health nurses and volunteers, health promoters, agricultural extension officers, non-governmental organisations and educators were identified as reliable sources for effective communication and productive engagement. The healthcare workers highlighted the need to explore the use of community volunteers as first responders at the community level.

*“Snakebite*, *like any other medical condition*, *needs prompt management to give a good prognosis*. *So creating an enabling environment is very important … is there any ambulance to carry these people*? *Are there volunteers to fall upon when they are bitten by the snake*? *Are there even numbers to even call the city*?*”* Female physician, healthcare workers FGD 2

The suggested channels of communication included community durbars, radio and television shows, religious platforms and community information centres. The use of validated local context-tailored infographics as community engagement tools was strongly advocated for by the community members. Local traditional authorities such as the assemblymen and women, the chiefs and religious figures as well as the traditional healers were identified as potential advocates who could serve as local champions to facilitate positive behavioural change.

*“… if you have snakebite prevention and management campaign posters placed at vantage locations that will help*.*”* Male, community members with snakebite history FGD 2

*“I think the chiefs; they have a say in most of these things*. *So if the chief is well informed and well educated and we make him understand the devastations snakebite is causing … it will trickle down to the community and help with complying with [control and preventive measures]*.*”* Male nurse, healthcare workers FGD 4

At least one participant in each of the 12 focus groups had lost a relative, friend or patient to snakebite in the past. The healthcare workers advocated for the prioritisation of snakebite at the national level to enhance and promote sustainable investment in snakebite prevention and control in endemic and at-risk regions. According to the healthcare workers, SBE is a health crisis that accounts for over 70% of hospital admission and mortality rates during farming seasons in rural communities.

*“Maybe they [policymakers] can attach the same seriousness they have for the tuberculosis programme*, *the HIV programme to snakebite because it is more like our HIV here*, *snakebite is more like our tuberculosis here*. *Because getting to the farming season*, *if you go to any unit we have over 18 patients on admission and you can count 12 on the account of snakebite*.*”* Male nurse, healthcare workers FGD 4

*“… the whole facility we had about 17 deaths and 16 were snakebite*. *That was when we weren’t having the anti-snake and they [patients] couldn’t afford*.*”* Male nurse, healthcare workers FGD 4

## Discussion

The needs and experiences of healthcare workers from various disciplines who contribute to the care of snakebite victims and community members with and without a history of snakebite were explored to identify the community-level snakebite research and management priorities. Our findings present similar research needs and challenges to those identified by the WHO’s strategy for the prevention and control of SBE but also introduce other research areas which indicate differences between the research community, healthcare workers and community members’ priorities.

The priority areas identified by the community members for further research and investigations include effective management of both acute and chronic effects of SBE and the need for context-tailored community engagement tools developed in consultation with community members, including chiefs and traditional healers. The healthcare workers, on the other hand, prioritised addressing health system challenges including training of health professionals, the development of a local standardized treatment protocol including pre-hospital care, and pre-clinical and clinical investigations on the efficacy of available antivenoms. Both community members and healthcare workers highlighted the need for scientific investigations into the efficacy of traditional medicines and collaborative strategies between traditional and allopathic treatment practices as priority research areas.

As part of efforts to generate the evidence needed to combat SBE, various research needs such as the epidemiology of the disease, basic studies on venom composition and mechanisms of action, pre-clinical and clinical studies, new therapies and improved diagnostics have been identified. Other areas highlighted include the development of targeted public health interventions, training programmes and the need for standardised treatment guidelines for clinicians [4,5,30–32]. In our findings, the community members prioritised treatment related challenges including access to health facilities, effective diagnostics, obtaining effective antivenom and cost of treatment as well as approaches to minimise both acute and chronic effects of the bite such as pain, management of the bite site, rehabilitation and mental health. The differences in the identified research priorities between the community members and healthcare workers and the available literature highlight the lack of follow-up by the formal health system once acute disease or envenomation has been reversed and victims are discharged. This corroborates our previous finding emphasising the need to pay more attention to the residual effects of SBE [2].

The concerns raised by both the community members and the healthcare workers on the varying efficacy of available antivenoms suggest that the WHO’s concern about the potential failure of antivenoms in the African market is appropriate. Advocacy for research into improving the potency, specificity and safety of current antivenoms, developing formulations with long shelf life and eliminating the need for a cold chain have been made by others [5,30,33]. Our findings indicate that, as healthcare workers prioritise the need for well-designed randomised controlled trials on antivenom efficacy, such studies could also integrate the evaluation of anaphylactic reactions and the development of long-term consequences as prioritised by the community members.

Exploring the efficacy of traditional medicines and strategies for collaborative treatment approaches between traditional and allopathic medicine was prioritised by both community members and healthcare workers. Several studies have documented potential antiophidic herbal products used by traditional healers in the treatment of snakebite in Africa, Asia and Latin America [34–40]. However, few *in silico*, *in vitro* and *in vivo* studies have been attempted on these products despite the identification of several secondary metabolites [41–46].

This study adds to the knowledge and calls for transdisciplinary SBE research while advocating for more grassroots engagement with community members and healthcare workers in the research agenda setting process. According to Gutiérrez *et al*., integrating a transdisciplinary approach to SBE research will promote the understanding of the context in which snakebites occur while advancing collaboration between scientists, clinicians and community-level actors such as local non-governmental organisations, local government and traditional authorities, and traditional healers [47]. This approach will facilitate a mutual learning process and a sense of involvement and ownership during the agenda setting and prioritisation processes and subsequently, promote the successful implementation of resulting interventions.

In the conduct of this study, we employed the services of native speakers of the local languages to facilitate and translate discussions between the researchers and the community members. However, the chances of losing some information during the translation process remain. To overcome this, the transcripts and audio files were further verified for accuracy by research team members who are native speakers of the local languages used during the FGDs. Although the study was conducted in two high prevalence regions with different geographic and socioeconomic conditions, there were no major differences in the identified priorities between the two regions. However, the findings may not represent the priorities of community members and healthcare workers in other districts of Ghana.

## Conclusion

The needs and experiences described at the community level underpin the importance of understanding the intricate sociocultural, political, economic and ecological contexts within which snakebite occur. Improving the health outcome and subsequent quality of life of victims require bringing the needs of community member and healthcare workers to the fore of the research agenda setting process. This will provide the needed leverage to bridge the gap between researchers, policymakers, clinicians and the local population. The participatory approach of engaging community members is a reliable way of improving health outcomes while increasing the acceptability and success of resulting SBE control and prevention policy interventions.
