# Correction: Community members and healthcare workers’ priorities for the control and prevention of snakebite envenoming in Ghana

**DOI:** 10.1371/journal.pntd.0011711

**Published:** 2023-10-23

**Authors:** The Neglected Tropical Diseases Staff

There are errors in the author affiliations. The correct affiliations are as follows:

Leslie Mawuli Aglanu^**1,2,3**^, John Humphrey Amuasi^**2,3,4,5**^, Evie Prokesh^**1**^, Alexis Beyuo^**6**^, Chrisantus Danaah Dari^**7**^, Sofanne J. Ravensbergen^**1**^, Melvin Katey Agbogbatey^**2,8**^, Austin Gideon Adobasom Anane^**2,4**^, Kabiru Mohammed Abass^**9**^, David G Lalloo^**10**^, Jörg Blessmann^**8**^, Benno Kreuels^**5,8**^, Ymkje Stienstra^**1,10**^

Affiliations

**1** University Medical Centre Groningen, Department of Internal Medicine/Infectious Diseases, University of Groningen, Groningen, The Netherlands

**2** Global Health and Infectious Diseases Research Group, Kumasi Centre for Collaborative Research in Tropical Medicine, Kumasi, Ghana

**3** Research Group Global One Health, Department of Implementation Research, Bernhard Nocht Institute for Tropical Medicine, Hamburg, Germany

**4** Department of Global Health, School of Public Health, Kwame Nkrumah University of Science and Technology, Kumasi, Ghana

**5** Division for Tropical Medicine, Department of Medicine, University Medical Centre Hamburg-Eppendorf, Hamburg, Germany

**6** Department of Development Studies, Simon Diedong Dombo University of Business and Integrated Development Studies, Upper West Region, Wa, Ghana

**7** Regional Health Directorate, Ghana Health Service, Upper West Region, Wa, Ghana

**8** Research Group Snakebite Envenoming, Department of Implementation Research, Bernhard Nocht Institute for Tropical Medicine, Hamburg, Germany

**9** Presbyterian Hospital, Agogo, Ashanti Region, Ghana

**10** Centre for Snakebite Research and Interventions, Liverpool School of Tropical Medicine, Liverpool, United Kingdom
